# Radiation Biology of Radiopharmaceuticals: Beyond External Beam Radiation Therapy

**DOI:** 10.3390/ph19040591

**Published:** 2026-04-07

**Authors:** Aeli P. Olson, Jonathan W. Engle, Mukesh K. Pandey

**Affiliations:** 1 Division of Nuclear Medicine, Department of Radiology, Mayo Clinic, Rochester, MN 55905, USA; olson.aeli@mayo.edu; 2 Department of Medical Physics, University of Wisconsin, Madison, WI 53705, USA; jwengle@wisc.edu; 3 Department of Radiology, University of Wisconsin, Madison, WI 53705, USA; 4 Mayo Clinic Comprehensive Cancer Center, Mayo Clinic, Rochester, MN 55905, USA; 5 Department of Molecular Pharmacology and Experimental Therapeutics, Mayo Clinic, Rochester, MN 55905, USA

**Keywords:** theranostics, radiopharmaceutical, radiobiology, alpha, beta, auger, radioisotope, radionuclide

## Abstract

The dynamic field of radiopharmaceuticals is currently experiencing an explosion of growth due in part to excitement over the emerging field of theranostics (therapy and diagnostics). Radiopharmaceuticals use physiological targeting methods to deliver radionuclides with medically relevant decay properties to disease biomarkers for diagnosis and treatment, offering opportunities for early disease imaging and radiation therapy treatment in disease pathologies that are inoperable or refractory to other forms of radiotherapy. Sustaining this rapidly growing field depends heavily on the continued design and production of novel, effective radiopharmaceuticals. Effective therapeutic radiopharmaceuticals cause complex and varied cellular responses, and to choose radionuclides that maximize therapeutic response, researchers must understand radiation biology. Cellular radiation response depends heavily on factors including linear energy transfer (LET), dose, dose rate, targeted location, direct or indirect energy deposition mechanisms, the broader cellular matrix, cellular stress signaling pathways, and endogenous radiation protection mechanisms. Because of the extensive application of low-LET external beam radiation on clinical cancer treatments, biological responses to low-LET form the basis of radiation biology and are generally considered transferable to high-LET radiopharmaceuticals. However, increased focus on high-LET, radiopharmaceutical therapy-specific radiation biology is motivated by differences between low- and high-LET radiation, external beam versus radiopharmaceutical therapy-induced biological response, and the observed varied clinical responses to radiopharmaceutical therapies. This review article summarizes historical understanding of low- and high-LET radiation responses within cells, with emphasis on radiopharmaceutical-specific responses when available, and discusses current gaps in understanding in the radiation biology of radiotheranostic pharmaceuticals.

## 1. Introduction

Radiopharmaceuticals use biological targeting vectors (e.g., peptides, hormones, signaling molecules, antigens, antibodies, small molecule inhibitors or substrates) tuned to cellular biomarkers (e.g., protein receptors overexpressed in cancers) to deliver medically relevant (therapeutic or diagnostic) radionuclides to diseased cells. In 1941, the first patient was successfully treated for hyperthyroidism with internalized radionuclides, specifically ^130/131^I [1], and in 1927, researchers used ^214^Bi to trace blood circulation [2]. Far predating these events, however, are the first treatments of patients with X-rays in 1896 [3] and the first reports of negative side effects from radiation exposure [1,4], marking the beginnings of the fields of X-ray external beam radiation therapy (EBRT) and radiation biology. EBRT and radiation biology are closely linked, and due to available technologies, radiation biology experiments throughout the 20th century primarily studied the impact of X-ray irradiation. Radionuclides incorporated into radiopharmaceuticals emit a range of particles with varying type, energy, dose rate, and intensity. Greater interest in radiopharmaceuticals has motivated the development of greater production, availability, and chemical incorporation strategies for a wider selection of radionuclides, each with unique emission profiles. These uniquely emitted radiation profiles elicit a complex series of responses within biological systems, and a thorough understanding of radiation biology can be leveraged towards designing more effective radiopharmaceuticals. Therapeutic radiopharmaceutical efficacy depends on delivering a sufficient therapeutic dose to diseased cells—a combination of the physiological targeting method and radionuclide decay properties. Radiation biology currently has the opportunity to expand beyond EBRT paradigms to include the study of radiopharmaceutical-specific responses, requiring careful consideration of the unique dose deposition profiles achievable from individual radionuclides. Important tools in this endeavor are radiation dosimetry and theranostics. Theranostic pairs match therapeutic drugs with diagnostic complements, identifying and stratifying disease, quantifying pharmacokinetics, and enabling dosimetric planning and post-therapy assessment. In some realizations, radioisotopic imaging analogs are employed, but radionuclides of differing elements in drugs with matched chemical properties can also be valuably exploited. Isotopic diagnostic analogs measure and quantify in vivo distributions of therapeutic radiopharmaceuticals for precise macroscopic dosimetry estimation and treatment planning [5]. A thorough understanding of radiation biology begins with first understanding the basic physical principles of particle emission and subsequent methods with which energy transfers to and is absorbed by matter.

### 1.1. Introduction to Types of Radiation Emitted from Decaying Radionuclides

Generally, the ratio of protons and neutrons within a nucleus determines the atom’s stability, with large nuclei decaying by splitting into nuclear fragments. The smallest emitted nuclear fragment is an alpha (α) particle, consisting of two neutrons and two protons. Neutron-rich nuclei decay through high-energy electron (β^−^) emission—a process which converts a neutron into a proton. Proton-rich nuclei decay by positron (β^+^) emission, electron capture, or internal conversion, which each convert a proton into a neutron. During electron capture decay, the process of proton conversion requires that the nucleus absorb an inner-shell orbital electron. Within internal conversion decay, an electron is ejected from the atom after dynamic electromagnetic multipoles within the excited nucleus interact with and transfer energy to an inner-shell orbital electron [6]. Within the emitted electron spectra, this internal conversion electron will have the highest energy. Internal conversion and electron capture decay result in removal of an inner-shell electron, creating a subsequent excited atomic electron state. As higher-energy electrons de-excite into lower-energy electron shells, the energy difference between shells is either reinvested to kick out an electron or emitted as a characteristic X-ray. When additional electrons are kicked out, more holes are created and more electrons can be emitted, resulting in a cascade of low-energy electrons [7,8,9] referred to as Auger [10] and Coster–Kronig electrons [11]. Excited atomic electron states de-excite through the competing processes of X-ray photon emission or Auger electron emission, and excited-state nuclei transition to lower energy states through the emission of gamma (γ) ray photons [8,9,12]. Three types of radioactive particles (emitted through four decay processes) are championed for their radiopharmaceutical therapy potential: α particles, β^−^ particles, and low-energy conversion and Auger electrons.

### 1.2. Introduction to Radiation Interactions with Matter for the Transfer of Energy

Fundamentally, radiation dose is the energy transferred to and deposited within matter, which occurs primarily through ionizations and excitations of atoms and molecules. Ionizations induced by the different types of radiation emitted from theranostic radiopharmaceuticals (primarily charged particles and photons) have significant consequences as they can disrupt chemical structures and produce chemically reactive free radicals.

#### 1.2.1. Charged Particle Interactions

The type and intensity of charged particle interactions with matter depends on particle size, charge, energy, and the approach towards the absorbing atom, namely the distance the charged particle’s trajectory is from hitting the absorbing atom’s nucleus. Charged particles interact with matter through electrical Coulomb forces, and two main classifications can be constructed, Coulomb interactions with the negatively charged orbital electrons of the absorber atom (Coulomb scattering) or Coulomb interactions with the positively charged nucleus of the absorber atom (radiative loss, also known as Bremsstrahlung radiation). Generally, Bremsstrahlung or radiative losses produce photons with energy high enough to leave the immediate location and thus do not contribute significantly to absorbed radiation dose.

Charged particle radiation from radiopharmaceuticals primarily transfers energy to matter through Coulomb scattering. In Coulomb scattering, an approaching charged particle has its kinetic energy reduced through repulsive Coulomb force from the charged particle it is approaching. After the incoming charge particle has lost its kinetic energy, the repulsive force will send it out at a new, deflected angle. When Coulomb forces of close approaching charged particles interact with orbital electrons, the strength of the interaction can result in energy transfer sufficient to kick an orbital electron out of the atom, producing an ion and free-electron pair. The amount of energy transferred from the incoming charged particle depends on the scattering angle, with maximum energy transfer occurring at a back-scattered angle of 180°. A soft collision describes ionization interactions between the charged particle and outer atomic orbital electrons, and hard collisions describe ionization interactions with inner atomic orbital electrons. The latter results in an ejected inner-shell electron, referred to as a delta ray (δ-ray), which has high energy such that it can induce additional secondary ionizations in surrounding atoms [4,8,9,12].

Generally, each of these energy transferring interactions is small, and charged particles undergo many scattering interactions, producing a tortuous and crooked path before completely transferring their kinetic energy to matter. Large charged particles are deflected at smaller angles than small charged particles, resulting in a straighter trajectory [4,8,9,12]. The rate of energy loss as charged particles move through matter is referred to as stopping power, expressed as energy lost per unit pathlength of the particle. For charged particles, stopping power can be broken down into radiative stopping power (Bremsstrahlung) and collisional stopping power (Coulomb scattering). Though statistical in nature, the coordinate vector sum of many small interactions is described as the charged particle’s range in a particular type of matter and is helpful in quantifying the average distances charged particles will travel in matter. Though related, the release of energy from a charged particle (stopping power) is different than the energy absorbed by matter as a charged particle travels through it (linear energy transfer) [4,8,9,12].

#### 1.2.2. Photon Interactions

Photons transfer energy to matter, which comprises electrons and atomic nuclei, through three main processes: the photoelectric effect, Compton scattering, and pair production. The likelihood of each process occurring, referred to as the interaction cross-section, is dependent on the atomic number of the encountered atom (Z) and the photon energy.

Dominating in likelihood at low energies (<30 keV in H_2_O) [13] and high Z, the photoelectric effect describes the absorption of a photon by an atom. Subsequently, the atom is excited to a higher electron energy state, or an orbital electron (most often from the K shell) is kicked out after overcoming the electron binding energy E_b_(K). To conserve energy and momentum, the atom (now ion) will have recoil energy; however, due to the significant mass difference between the ion and electron, the recoil energy is extremely small, and the kinetic energy of the freed photoelectron is estimated as the energy of the photon subtracting the binding energy E_b_(K). The photoelectric effect produces an inner-shell electron hole and excited atomic state, resulting in subsequent emission of either X-Ray or Auger electrons [8,9,12].

Similar to a collision, a photon can scatter off and transfer energy to an electron through the process of Compton scattering, which is the most common process occurring within tissues at medically relevant energy ranges (30 keV–24 MeV in H_2_O) [13]. The fraction of energy transferred to the electron depends on the photon energy and the scattering angle. In the non-ionizing, low energy, visible light range, photons scatter off electrons, changing directions but not transferring energy (Raleigh or Thomson scattering). Generally, it is conceptualized that, in Compton scattering, photons scatter off outer-shell electrons, kicking the electrons out of their atomic confines and producing ionizations and free radicals [8,9,12].

When a third particle is involved, photons can split into a pair of an electron and a positron. In nuclear pair production, the extra particle is the nucleus of an atom, and in triplet pair production, it is an electron. As the energy of the photon is converted into the rest mass of the positron and electron, a photon must have minimum energy of 2m_e_c^2^ (1022 keV), and pair production dominates in likelihood above 24 MeV in H_2_O [13]. Any energy above the 1022 keV threshold is transferred to particles as kinetic energy. After pair production, the electron and positron will move through matter, transferring energy via collisions and bremsstrahlung radiative losses until the positron annihilates with an electron [8,9,12].

#### 1.2.3. Neutron Interactions

Though rarely emitted from radionuclides, neutron interactions are important in radionuclide production and are well described by classical mechanics, governed mainly by elastic and inelastic collisions with nuclei. Due to similar masses and classical collisional mechanics, the greatest amount of energy is transferred from a neutron to matter per interaction with a hydrogen nucleus (the recoil nucleus). Materials with large elemental fraction hydrogen, such as H_2_O, are the best materials to absorb energy from neutrons (also referred to as neutron moderation). Collisions of low-energy neutrons can result in nuclear absorption, resulting in a nuclear reaction [4]. Because neutrons are not charged, they do not directly produce ionizations. Their interactions with atomic nuclei (through absorption or collision recoil energy transfer) generate excited nuclei, which emit secondary radiation, including photons or charged particles [8].

#### 1.2.4. Important Concepts Regarding Radiation Interaction and Absorbed Dose

When considering the radioactive particles and energy ranges used medically, radiation is most likely to transfer energy to matter via ejecting electrons from atoms [8,9,12]. The subsequently produced ionizations and free radicals can be disruptive to surrounding chemical processes and moieties (e.g., DNA, proteins, and other biological structures). Linear energy transfer (LET) measures the energy absorbed by matter from radiation per unit pathlength the radiation traverses. From high-LET (>1 keV/mm) radiation (α particles and low-energy electrons), energy is absorbed within a short range around the site of decay. When delivered to diseased cells, shorter-range radiation would result in delivery of the emitted particle’s energy with less off-target radiation dose, minimizing the side effects. This would increase the therapeutic window, allowing administration of a higher, more cytotoxic radiation dose and potentially better treatment outcomes. Low-LET (<1 keV/mm) radiation (γ, X-ray, and β^−^) travels longer distances, providing more homogenous dose distributions despite inhomogeneously distributed biological targets but increasing dose to off-target tissues. Observed differences in cell-killing efficacy from differing LET radiations motivated the development of the relative biological effectiveness (RBE) metric, which is determined for each emitted particle type. Experimental RBE measurement traditionally occurs by irradiating cell lines with multiple dosages of a radiation type and normalizing the test radiation type (commonly the dose which achieves 37% cell survival) to that of the reference photon emitter dose (e.g., ^137^Cs, ^60^Co, and 250 keV X-ray) achieving the same survival outcome [14,15,16,17]. Different particles have different LETs, which lead to differing radiobiological responses comparably quantified by differing RBE. Often overlooked in the discussion of radiopharmaceutical radiation biology is the impact of the decay event and daughter nucleus recoil.

Using conservation of momentum and energy, the masses of atoms and particles involved, and measured energies of released particles, the recoil energy of the daughter can be computed. For radionuclides relevant to radon (^222^Rn) decay, researchers determined that the energy of the recoil nuclei involved in α decay contributed 2% of calculated dose and β^−^ decay contributed 0.0001% [18]. With α particles having higher energy and more similar mass to their daughter nuclei than other radioactive emissions, a greater portion of energy remained with the daughter nuclei post α decay (0.08–0.17 MeV) compared to β^−^ decay (0.18–18.67 eV) [18]. When the energy of the recoil nuclei is greater than the chemical bonds constraining it, the daughter nuclei can dislodge from the targeted pharmaceutical and redistribute throughout the body based on the nucleus’ elemental biochemistry. If the recoil energy is less than the energy of chemical bonds and the parent and daughter have similar elemental chemistry, researchers have shown that the daughter remains confined within the chelator and chemically attached even after decay [19,20]. In a ^90^Sr/^90^Y generator system, 99.9% of ^90^Y atoms remain confined within the DOTA chelator post-decay of its coordinated parent [^90^Sr]Sr-DOTA [20], yet it is well established that ^225^Ac’s α decay results in the release of radioactive daughters [21]. The impact of nuclear recoil is less important in cases with a radionuclide decaying to a stable daughter.

## 2. Differences Between External Beam Radiation and Radiopharmaceutical Therapy

Because low-LET EBRT is extensively applied to clinical cancer treatments, biological responses to low-LET radiation lay the foundation of radiation biology. Knowledge referencing low-LET radiation response is considered transferable to high-LET radiopharmaceuticals; however, differences between low and high LET radiation as well as radiation delivery methods (external beam versus radiopharmaceuticals) motivate specific focus on radiopharmaceutical therapy-induced biological response [22]. Differences between EBRT and radiopharmaceuticals are summarized in Table 1, with discussion of potential clinical implications. There are concerns that low-dose-rate radiation allows for repair of sublethal damage, but dose response characterization for therapeutic radiopharmaceuticals is not well understood [23].

## 3. Radiation Biology Effects

**Figure 1:**
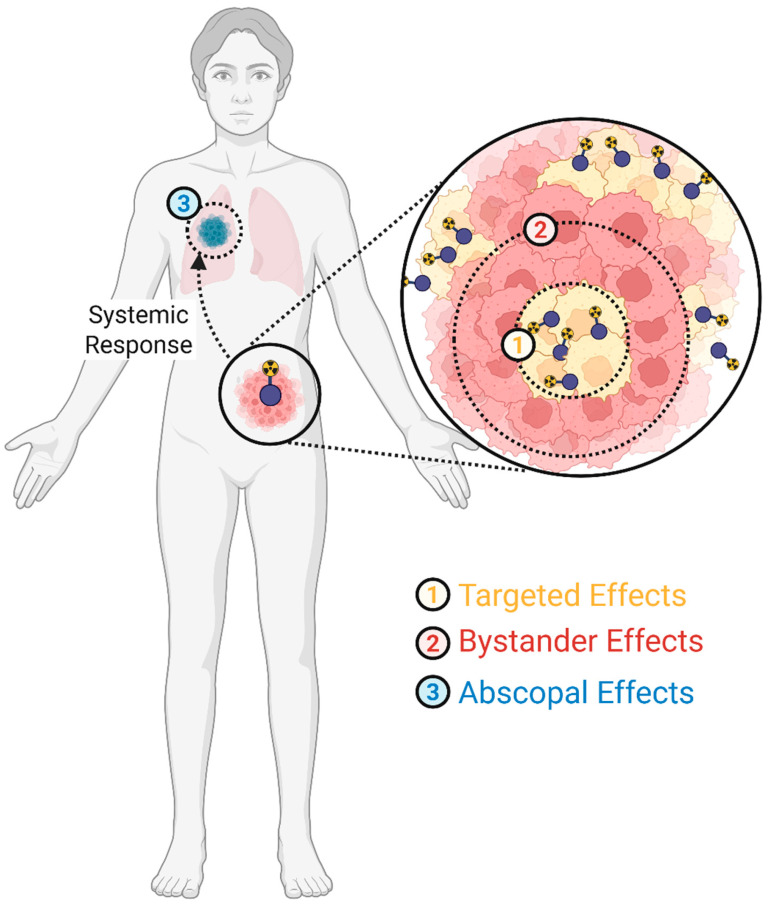
Systemic radiation response throughout the body described in terms of targeted, non-targeted bystander, and abscopal effects. Created in BioRender. Olson, A. (2026) https://BioRender.com/sc3603b, accessed on 26 March 2026.

Radiation from therapeutic radiopharmaceuticals causes both targeted and non-targeted biological effects. Targeted radiation effects include damage to cell structures, which in turn causes decreased cellular function. Non-targeted radiation effects involve cellular responses to radiation through the activation of various complex stress signaling pathways that can cause cell death for irradiated cells and surrounding non-irradiated cells (bystander effect) [22,24,25]. Both targeted and non-targeted effects depend upon dose, dose rate, dose fractionation, and LET. The cell-killing efficacy of both effects increases with increased LET, motivating the use and development of high-LET radiopharmaceuticals. Occasionally documented from X-ray or proton beam therapy, abscopal effects describe systemic changes in disease at distal, un-irradiated locations within the body, resulting from localized irradiation [26,27,28,29]. Figure 1 illustrates these three observed effects. Additionally, tissue radiosensitivity is impacted by cell proliferation, tissue oxygenation (impacting free radical generation), the volume of tissue irradiated, and tissue microenvironment (impacting bystander and abscopal effects, discussed later) [17].

### 3.1. Targeted Radiation Biology Effects

#### 3.1.1. Targeting DNA Damage

Regarding radiation dose deposition, two main mechanisms, direct and indirect, help frame induced chemical damage. Direct mechanisms denote energy deposition from radiation to cellular structures. Each interaction of radiation with matter ejects single electrons from atoms within those structures, subsequently causing ionization and disrupting essential biological structures and critical cellular functions. Indirect mechanisms denote energy transfer from radiation to ionic radical species (most commonly water), which create chemically reactive intermediaries. When ionized, water forms reactive oxygen species (ROS), hydroxy free radicals, and hydrogen peroxide (H_2_O_2_). These radicals and additional reactive nitrogen species (RNS) can react destructively with cellular structures. Radiation-generated free radicals, ROS, and RNS are chemically indistinguishable from corresponding endogenously generated species created through oxidative stress and cellular metabolism. Indirect mechanisms act across longer distances than direct mechanisms within the cell, but indirect mechanisms have greater susceptibility to electron recombination and neutralization through endogenous antioxidants and ROS cellular protection mechanisms [17,22,25]. Within the cell, DNA is the most sensitive subcellular structure to radiation, and, in general, effective cell killing is optimized by maximizing the radiation dose delivered to the cell nucleus and thereby to the DNA [15,17], with protein and lipid oxidation offering alternative routes to cell death [22].

Single-strand breaks (SSBs), double-strand DNA breaks (DSBs), base deletion, modification, crosslinking, and multiple damage sites (MDSs) are forms of DNA damage observed after radiation exposure. The extent of radiation-induced DNA damage is dependent on the LET of radiation administered, absorbed radiation dose, and dose rate. MDS, also referred to as clustered DNA lesions, describes at least two DNA lesions (e.g., base modification, SSB, and base crosslinking) caused by the same decay event which are positioned within two DNA helix turns. DSBs are the lowest form of MDS complexity [30,31]. MDSs have low endogenous production, are indicative of ionizing radiation, and increase in complexity and quantity when caused by higher-LET radiation. Primarily, high-LET-induced MDSs are repaired through homologous recombination pathways [30], and their enhanced toxicity is attributed to increased repair difficulty [22]. Moreover, increased difficulty in repairing MDSs prolongs the lifetime of the lesion and, depending on the cell replication phase, increases the mutation rate. Increased mutation rates, chromosomal aberrations, translocations, and sister chromatid (identical copies of a chromosome formed during DNA replication) exchanges all result from the increased complexity of MDSs caused by exposure to high-LET radiation [30]. Important to DNA damage is a cell’s capacity to repair said damage. Thorough reviews into DNA damage repair mechanisms [32], radiation-induced DNA damage repair [33], and radiopharmaceutical-specific DNA damage response [34] are available.

High-LET radiation induces significant DNA damage through both indirect and direct mechanisms compared to low-LET, which primarily acts through indirect mechanisms and produces lower complexity DNA damage. Cell culture experiments in the antioxidant dimethyl sulfoxide (DMSO) determined that low-LET radiation induces SSB [35], primarily via indirect mechanisms (70%) [22,30]. With low-LET radiation, hypoxia can cause decreased radiosensitivity due to decreased oxygen availability for ROS generation [4]. The proportion of direct to indirect contribution increases with increased LET [22,30], and high-LET cell killing is less impacted by hypoxic conditions [4]. High-LET radiation acts upon DNA with significant direct contribution, creating greater DSBs and MDSs [35]. More complex DNA damage induces cell death with greater efficiency, motivating use of high-LET over low-LET radiation in therapeutic radiopharmaceutical design.

#### 3.1.2. Targeting Other Critical Cellular Structures

In response to radiation exposure, cell membrane and morphology changes include membrane ruffling, the development of surface blebs, changes in the size and number of microvilli, and membrane retraction [36]. Protein hydroperoxides and lipid peroxides formed through chemical reactions between free radicals and cytoplasmic proteins or cell membrane lipids can both initiate oxidative chain reactions, which multiply the destructive effects of the first radical [22,25]. Direct radiation interactions with lipids and the resultant lipid peroxidation increase membrane permeability, which disrupts transmembrane processes, damages transmembrane protein function, disrupts ion gradients, and changes membrane fluidity [25]. Upon exposure to radiation, the glycocalyx (a dense matrix of carbohydrates surrounding the outside of the cell membrane) experiences reversible, dose-dependent changes to charge, membrane-bound calcium, receptor expression, receptor affinity, and enzyme distribution and activity [36]. A widening of intercellular spaces and functional changes to gap junctions impact cell communication [36]. Radiation also affects mitochondrial DNA; however, the effect of induced damage on cell survival is poorly understood [25], especially within the context of radiopharmaceuticals.

Similar to nuclear DNA, mitochondrial DNA experiences base deletions [37], strand breaks [38], and base mismatches [39]. In response to external photon irradiation, researchers observe disrupted mitochondrial function, impacted membrane potential, altered oxidative phosphorylation, damaged electron transport chain complexes, decreased ATP synthase, and increased ROS production [40]. Using X-ray irradiation, multiple studies have found a significant radioprotective effect of manganese superoxide dismutase [41,42,43], an antioxidant specific to ROS neutralization within the mitochondria [44]. α particles and carbon ion microbeams cause mitochondrial depolarization and fracture, impacting mitochondrial function and inducing pro-apoptosis signaling through release of cytochrome C [45]. Irradiation of mitochondria causes functional damage, which impacts metabolism and, most importantly, causes prolonged production of the radical superoxide, which contributes to latent cell stress. After irradiation, cell mitochondria increase in mass and quantity [46,47], explained through speculated compensation for decreased function, specifically energy production due to damage to the electron transport chain [40]. Much of the study into the impact of ionizing radiation on mitochondria is exclusively from various high-dose (>1 Gy), low-LET external beam irradiation studies.

### 3.2. Off-Target Radiation Biology Effects

#### 3.2.1. Activating Stress Signaling Pathways and Bystander Effects

Non-targeted radiobiology effects comprise observed cytotoxic cellular responses not resulting from radiation damage to cell structures that can also result in DNA damage, mutations, apoptosis, cell death, and chromosomal aberrations [22,25]. These effects, which depend on LET, dose, and dose rate [25], are associated with activation of pro-apoptotic and oxidative stress cell signaling pathways. These signaling pathways transmit to neighboring cells via secondary molecular messengers, including ROS, ATP, RNS, Ca^2+^ ions, and cytokines [22,24,25,45], leading to ROS induction in non-irradiated cells [25]. Numerous experiments illustrate these curious bystander effects through media transfer experiments in which researchers dose cells with therapeutic radiopharmaceutic agents, remove and wash radiopharmaceutical media, incubate irradiated cells in clean media, and transfer that media to non-irradiated cells. Researchers observe substantial cytotoxicity in un-irradiated cells [24,48,49,50,51,52,53,54,55,56,57,58], including the production of DNA lesions [24,49,53,56,58] after initial radiopharmaceutical localization to either cellular membranes or cell nuclei [24].

Irradiation of cellular membranes with high-LET particles activates ceramide production, a secondary messenger of cell apoptosis, and triggers cell death through the formation of lipid rafts, which change membrane structure and fluidity [25]. In conjunction with adjusted membrane fluidity, lipid rafts activate protein kinase B (AKT), mitogen-activated protein kinase (MAPK), extracellular signal-regulated kinases 1 and 2 (ERK1/2), c-Jun N-terminal kinase (JNK), and p38 kinase pathways, contributing to DNA damage and helping compensate for less effective non-nuclear targeting [24]. Irradiation of the cytoplasm alters protein synthesis and mitochondrial oxidative phosphorylation, promoting oxidative stress [25].

Within media transfer experiments, irradiated donor cells experience cytotoxicity from both targeted and bystander effects, but unirradiated recipient cells experience cell killing from bystander effects. Using this logic, researchers calculated the bystander contribution to toxicity by subtracting recipient cell survival fraction from 100%. Next, they calculated the targeted toxicity contribution by subtracting both the corresponding bystander toxicity and donor cell survival fraction from 100% [24]. When delivering ^125^I-labeled compounds to the cellular membrane, cytoplasm, and nucleus, targeted effects from high-LET Auger electrons accounted for 13.2%, 12.8%, and 50.7% of induced cell death for each location, respectively. Between nuclear and cell membrane targeting, researchers measured comparable non-targeted cell death [24], illustrating the substantial contribution of non-targeted effects towards cell death independent of nuclear targeting. Because of this observation, non-targeted strategies should be considered during therapeutic radiopharmaceutical development. In the same publication, ex vivo studies exhibited peripheral tumor accumulation of an ^125^I compound, yet showed uniform, homogenous distribution of DNA with DSBs, likely due to high-LET radiation non-targeted bystander effects [24].

**Figure 2:**
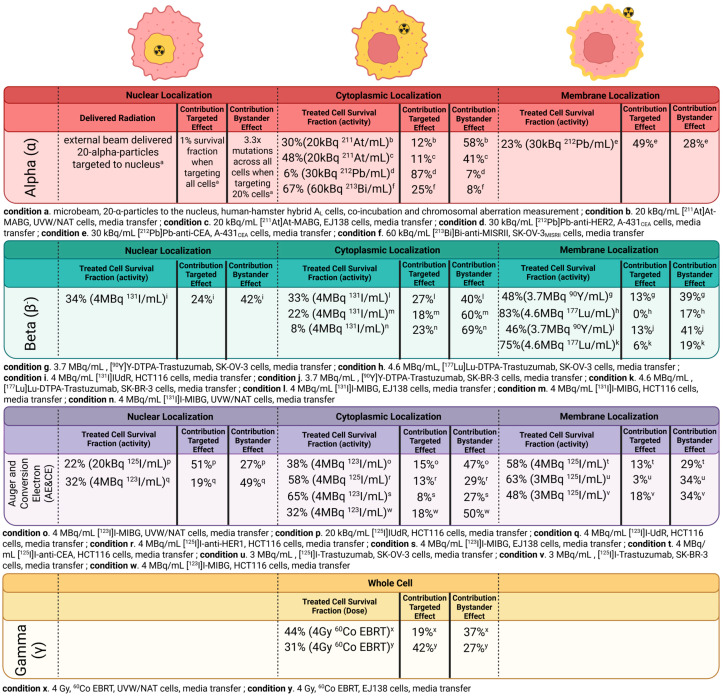
Schematic depicting calculated fractional contribution of targeted and non-targeted bystander effects towards cellular radiation toxicity. Targeted/bystander contribution calculation method adapted from [24] with data extracted from donor and recipient cell survival curves in media transfer experiments [24,55,57,59,60,61]. Conditions are summarized in lettered footnotes (e.g., a, b, c) beneath each emission section and can be found in the following references: a [61], b, c, l, n, o, s, x, and y [57], d, e, and f [59], g, h, j, k, u, and v [60], i, m, q, and w [55], p, r, and t [24]. Created in BioRender. Olson, A. (2026) https://BioRender.com/kbex7ny, accessed on 26 March 2026.

Applying the method of targeted/bystander contribution calculation, cytotoxic contributions from targeted and non-targeted bystander effects were calculated from approximated survival fractions extracted from published survival curves of media transfer experiments [24,55,57,59,60]. Figure 2 depicts the approximated contributions for differing types of therapeutic emissions and cellular targeting locations. As much as possible, similar administered activities were used for easier comparison. No appropriate media transfer study targeting the cell nucleus with an α-emitter was found, so details from a study of genomic instability following nuclear targeting with an alpha particle beam are included [61]. Within α-emitter targeting, the contribution of targeted and bystander cytotoxicity can change dramatically depending on the specific radionuclide (^211^At, ^212^Pb, and ^213^Bi) implemented, with ^212^Pb studies reporting the highest proportion of targeted cytotoxicity. Interestingly in Figure 2, conditions b (α, cytoplasm), n (β^−^, cytoplasm), o (AE/CE, cytoplasm), and x (γ, whole cell) match those of c, l, s, and y, respectively, changing only the cell line. Conditions c, l, s, and y reliably experience poorer cell killing and a lower bystander effect contribution to toxicity. For radiopharmaceutical-specific conditions (c, l, and s), this can be explained through the significantly lower uptake of [^131^I]I-MIBG in EJ138 cells as compared to UVW/NAT cells reported [57]. Though equal radioactivity was administered to each cell type, differences in radioactivity uptake (likely due to differences in receptor expression) result in differing absorbed radiation dose and cell survival. Because of cellular complexity and variable response, it is important that radiopharmaceutical-specific radiation biology research implements standardized techniques to determine and report findings, particularly in terms of cellular absorbed dose. For EBRT conditions x and y, condition y achieved greater cell killing with a lower contribution from the bystander effect, showing the difference in sensitivity, activation, and response of different cells to the same radiation type and dose. The impact of emitted electron spectra is seen by comparing conditions p and q in Figure 2, which both include the delivery of an Auger and conversion electron to the nucleus of the same cell line. In q, 4 MBq/mL [^123^I]IUdR achieves worse cell killing than condition p with 20 kBq/mL [^125^I]IUdR despite 20× greater activity.

#### 3.2.2. Abscopal Effects

It is hypothesized that abscopal effects are primarily mediated by the immune system. Radiation interacts with the immune system in a variety of manners, including immunogenic cell death—a process which initiates adaptive immunity of the host against antigens expressed on the dying cells [62]. Within the tumor microenvironment (TME), EBRT stimulates the release of pro-inflammatory cytokines (e.g., IL-1β, TNF-α, and IL-12) and upregulates tumor antigen presentation [62]. Dendritic and CD8+ effector T cell activation is further promoted through EBRT-stimulated release of extracellular ATP, High-Mobility Group Box 1 (HMGB1) protein, and surface calreticulin [63]. In addition, radiopharmaceutical therapy stimulates cell surface expression of surface annexin A1 and type I interferon (IFN-1) secretion [64,65,66]. Cells surviving radiopharmaceutical therapy have phenotypic changes, including major histocompatibility complex-I (MHC-I) expression (important in CD8+ cytotoxic T lymphocyte cell killing) and increases in leukocyte antigen expression, among others [65]. Case studies have documented rare reductions in distal metastasis after patients receive EBRT localized to primary tumors [26,27,28,29]. Combining radiation with immune checkpoint inhibitor immunotherapy holds promise in increasing these abscopal effects [67]. Radiopharmaceutical therapy, specifically, strongly modulates both innate immune populations and adaptive immune response, particularly when administered in conjunction with immunotherapies. Yet, the effects of particle type and dose on these immune responses are not fully understood [65].

## 4. Therapeutic Radionuclides

The three particle classifications mentioned above (α, β^−^, and conversion and Auger electrons) are championed for their therapeutic potential, with significant intra-class variation in emitted particle profiles from each radionuclide. When designing therapeutic radiopharmaceuticals, careful attention needs to be given towards radionuclide choice. Table 2 summarizes emitted particle energies, LETs, ranges, and approximate RBE. To deliver high radiation dose to a tumor, high RBE is desired. Pharmacokinetically targeted, high-RBE, high-LET radionuclides have immense potential within radiopharmaceutical therapy to eliminate micrometastases [16,68,69]. Of decay radiation, α particles have the highest LET, highest RBE, and ranges on the order of multiple cell diameters, providing a powerful therapeutic tool to maximize decay energy absorption to a targeted location. Though having high LET and high RBE, Auger and conversion electrons can have short ranges, making them frequently sensitive to subcellular distribution. Short-range, high-LET radiation can provide specificity in cell killing while sparing nearby adjacent cells, decreasing radiation therapy side effects. Finally, low-LET β^−^ particles have long ranges in tissue, increasing the dose to off-target tissues. Most observed cellular responses to radiation increase with increasing LET, demotivating the application of low-LET β^−^-emitting radionuclides. However, β^−^-specific advantages exist.

### 4.1. β^−^-Specific Radiation Biology Considerations

Clinically approved, low-LET, low-RBE, β^−^-emitting radionuclides (e.g., ^177^Lu, ^90^Y, ^131^I, and ^161^Tb) with pathlengths as long as 11 mm in water [73] deposit a lower dose compared to high-LET particles and can experience dose range (cross-fire) effects [74]. These cross-fire effects result in an absorbed dose to the targeted cell (self-absorbed dose) that is lower with β^−^ particles compared to higher LET particles. Additionally, cross-fire results in more uniform dose distributions across tumors than high-LET radionuclides, suggesting that β^−^-emitters are better suited for large tumors than micrometastasis [35].

**Figure 3:**
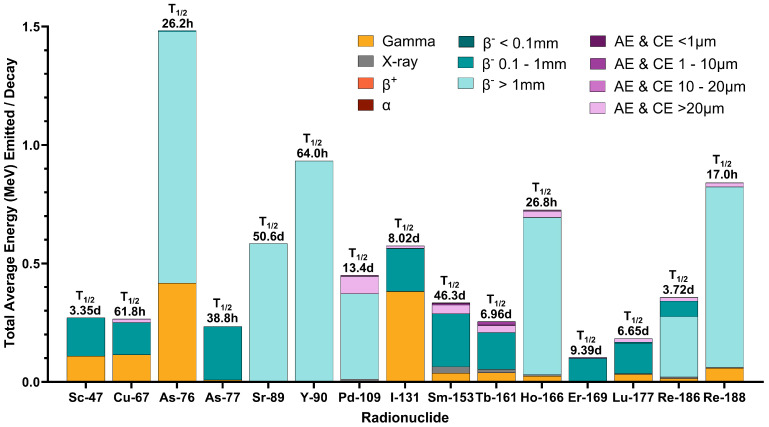
A comparison of total average energy (MeV) emitted by some β^−^-emitting radionuclides per decay, stratified by emission type and electron range with annotated half-life. Electron ranges were calculated from fitting continuous slowing down approximation ranges (R_CSDA_) in water reported and calculated from NIST [73] and GEANT4 Monte Carlo simulation [75]. Nuclear decay properties are sourced from [76,77].

The absorbed radiation dose delivered by therapeutic radiopharmaceuticals can be limited by biological factors (e.g., low target receptor expression, poor vasculature and blood delivery, hypoxia, and endogenous protection factors), pharmacologic and pharmacokinetic factors (e.g., slow tumor uptake, fast tumor clearance, low binding affinity, and low apparent molar activity), and radiologic factors (e.g., emissions, low LET, short radionuclidic half-life, and high dose to normal tissues). Some of these features can be tuned through radiopharmaceutical design, but LET is fixed by radionuclide choice. Similar to Auger-emitting radionuclides, emitted β^−^-energy spectra impact the absorbed dose. Differences in emission profiles for various β^−^-emitting radionuclides are presented in Figure 3.

**Figure 4:**
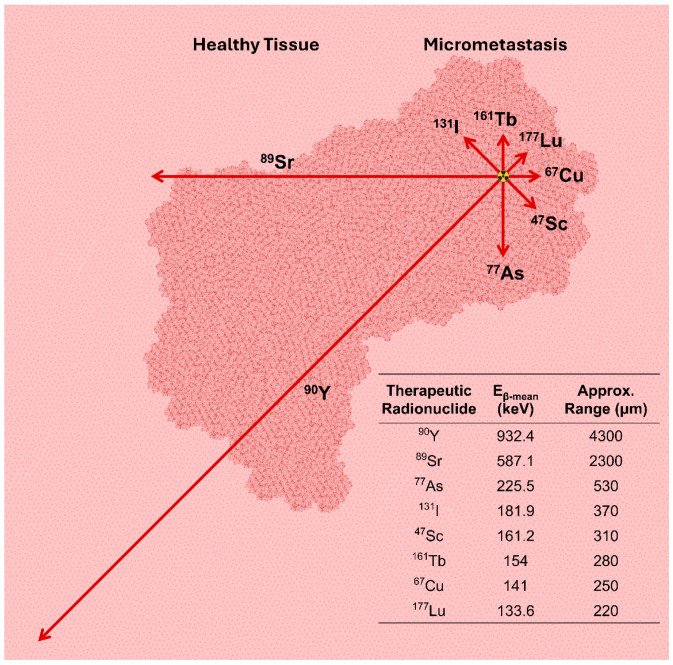
Illustration allowing a comparison of approximate distances mean β^−^ emissions travel from the point of decay for common therapeutic β^−^-emitting radionuclides. Nuclear decay properties are from [76,77], and electron ranges were approximated by fitting R_CSDA_ from NIST [73] and GEANT4 Monte Carlo simulation [75] in water. Created in BioRender. Olson, A. (2026) https://BioRender.com/cujxvtw, accessed on 26 March 2026.

To better illustrate the impact of emitted electron spectra, Figure 4 provides a comparison of approximate ranges for the average energy of emitted β^−^ particles, illustrating the differences in electron range from common β^−^-emitting radionuclides. The longer range of ^90^Y’s average energy emission provides opportunities for dose range effects to produce more homogenous dose distributions; however, it limits how tightly dose can be administered and off-target dose avoided. A group comparing cellular-level dose deposition from β^−^-emitting radionuclides found that within a 100 μm sphere, 71% of ^161^Tb’s absorbed dose (44.5 Gy) came from Auger and conversion electrons, and far greater dose deposition was achieved as compared to similar conditions applying ^177^Lu (24.5 Gy), ^67^Cu (24.1 Gy), ^47^Sc (14.8 Gy), or ^90^Y (1.36 Gy) [78,79]. Researchers compared bone marrow dose from three prominent, preclinically and clinically developed β^−^-emitting radionuclides with prominent differences in emitted electron spectra, which are reprinted in Table 3. Outside of the bone marrow, with ^90^Y’s high energy, 932.4 keV β^−^ emission accounted for a significantly higher (65–80%) radiation dose (as quantified via active bone marrow S-values) than ^177^Lu and ^161^Tb’s medium energy emissions. The MIRD schema for computing internalized radionuclide dosimetry uses S-values to quantify the absorbed dose per decay. All computed S-values for ^161^Tb were higher than ^177^Lu, illustrating the larger therapeutic punch offered by ^161^Tb’s high-LET Auger and conversion electrons. The differences between ^161^Tb and ^177^Lu active bone marrow S-values decreased with increasing source distance, indicating greater source distribution dependency on dose deposited by ^161^Tb over ^177^Lu [80].

Despite dose rate limitations, β^−^-emitters lead in radiopharmaceutical therapy application due to scalable production using neutron bombardment within nuclear reactors [81] and more developed chemical incorporation strategies [82]. The VISION trial, which implemented [^177^Lu]Lu-PSMA-617 for the treatment of castration-resistant metastatic prostate cancer, measured an increase in progression-free survival from 3.4 months to 8.7 months and an increase in overall survival from 11.3 months to 15.3 months. Of patients in the treatment group who had measurable lesions, 9.2% experienced complete response and 41.8% a partial response [83]. The NETTER-1 trial, which used [^177^Lu]Lu-DOTATATE to treat midgut neuroendocrine tumors, noted that 1% of patients experienced a complete response and 17% experienced a partial response [84]. When implementing [^177^Lu]Lu-DOTATATE treatment at an earlier disease stage (NETTER-2 trial), progression-free survival was increased from 8.5 months to 22.8 months, 5% of individuals experienced complete response, and 38% experienced a partial response [85]. In clinical trials, β^−^-emitting ^177^Lu radiopharmaceutical therapies measure tumor response some cases of advanced disease, but to increase treatment response, deeper understanding in radiopharmaceutical-specific radiobiology and tumor biology is necessary.

Recent work applying EBRT principles of absorbed dose response to therapeutic ^177^Lu clinical trial data to predict tumor response developed a threshold technique, correlating absorbed dose from the radiopharmaceutical treatment course to changes in tumor volume. By visually inspecting data, three regions could be delineated for three retrospective neuroendocrine tumor clinical trials. The lowest absorbed dose region (<~100 Gy) had unpredictable response, the next highest region (~100–150 Gy) experienced tumor control (<20% increase in tumor volume), and the highest region (>~150 Gy) experienced tumor control without substantial gains for additional radiation dose. This task group determined that heterogenous absorbed doses provided varied response, with some lesions having the same absorbed dose experiencing differing tumor control responses and vice versa, indicating additional implications from variables (e.g., tumor biology) currently overlooked in clinical trial design and radiation response [86].

### 4.2. α-Specific Radiation Biology Considerations

**Figure 5:**
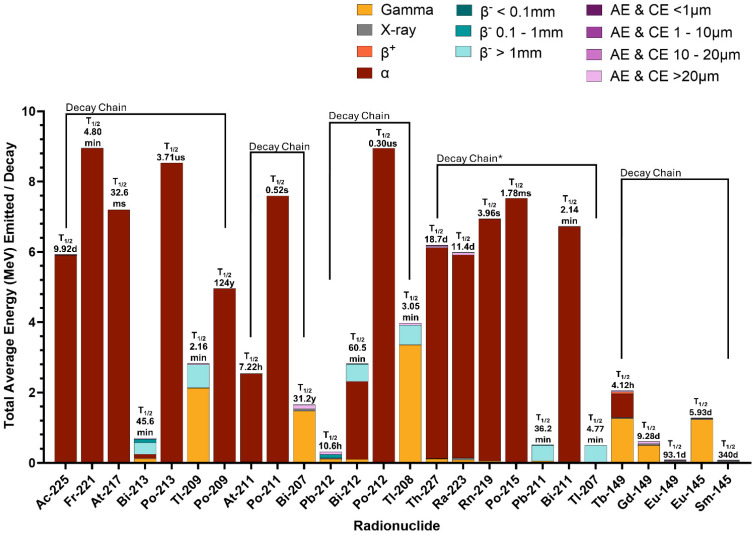
A comparison of total average energy (MeV) emitted by common alpha-emitting radionuclides per decay, stratified by emission type and electron range with annotated half-life and grouped by decay chains. Nuclear decay properties are sourced from [76,77]. * ^227^Th decay chain also includes ^211^Po.

Of decay radiation, alpha (α) particles achieve the highest RBE; however, α-emitters often decay to unstable daughter radionuclides, which provide confounded dose distributions as recoil energy decouples daughters from molecular constructs [87]. The average energies per decay from several α-emitting radionuclides chosen for radiopharmaceutical therapy, grouped via decay chain, are presented in Figure 5. Recoil significantly impacts ^225^Ac and ^212^Pb radiopharmaceutical therapy as the free ^212/213^Bi (t_1/2_ = 60.5/45.6 min) daughter redistributes to the kidneys, causing significant off-target kidney dose [88]. With each β^−^ decay of a ^212^Pb atom, the α-emitting radioactive daughter ^212^Bi releases from the chelator DOTA in 36% of occurrences [89] and dissociates from TCMC in 30% [90]. Strategies to minimize toxicity from ^212/213^Bi renal accumulation include metal chelation therapy to scavenge unbound ^213^Bi [91], ^225^Ac/^213^Bi generator and subsequent radiopharmaceutical development with stably complexed ^213^Bi [92], encapsulating ^225^Ac and recoiling daughters in nanoparticles [93], and localized administration of α-emitters into solid tumors [94].

Many researchers hypothesize that unbound ^213^Bi migrates to the kidneys from ^225^Ac, decaying within the blood pool as opposed to redistributing from ^225^Ac targeted to the tumor. Using a fast, pharmacokinetically targeted ^225^Ac-labeled PSMA small molecule, less ^213^Bi activity accumulated within the kidneys as compared to the slow blood pool clearing ^225^Ac-labeled 7.16.4 antibody [95]. Additionally, five patients received post-[^225^Ac]Ac-PSMA-617 SPECT imaging, tracking the biological distributions of ^221^Fr (t_1/2_ = 4.80 min) and ^213^Bi daughters. Researchers measured significant differences between the effective half-lives of daughters within the kidneys and insignificant differences between the effective half-lives of the daughters in metastatic lesions, further supporting the idea that ^213^Bi does not redistribute from the tumor but primarily from ^225^Ac radiopharmaceuticals decaying within the blood pool [96]. The concern of daughter redistribution has motivated study and application of the α-emitter ^211^At, which has a shorter, more dosimetrically simple decay chain [97].

The majority of α-emitter productions require extremely high energy particle accelerators, high intensity, heavy ion beams, radioactive mass separators, or generators constructed from controlled reactor products. These barriers currently limit global production capacity and impede availability [1,98,99,100,101]. Even so, clinical trials are growing in interest, as studies have shown the increased effectiveness in tumor control when comparing α versus β^−^ radiopharmaceutical therapies [92,102,103,104,105], with high-LET α-emitters even overcoming resistance to low-LET β^−^ radiopharmaceutical therapy [106]. In recent phase II clinical trial results, [^212^Pb]Pb-DOTAMTATE achieved 54.3% response in radiopharmaceutical therapy treatment-naïve patients, 30.8% partial response patients previously treated with ^177^Lu-labeled somatostatin analogs, and a 96.2% disease control rate in ^177^Lu treated patients [107]. When comparing α-emitting ^225^Ac and β^−^-emitting ^177^Lu, ^225^Ac caused 700× more DSBs and resulted in 60% more cells in G2/M cell cycle arrest [104]. In murine prostate cancer models, [^225^Ac]Ac-NM600 induced stronger immunomodulatory effects, including activating effector and memory T cells, depleting suppressive Tregs, and elevating pro-inflammatory cytokines and chemokines higher than [^177^Lu]Lu-NM600 [103]. Comparisons can also be made between α-emitters. Being earlier in the decay chain, ^225^Ac radiopharmaceutical therapy provides three additional α-particles than the ^213^Bi daughter. Researchers illustrated the impact of these additional α-particles in tumor control in a rat breast cancer model by showing a significant increase in survival when dosing with a similar number of total decays. The median survival increased from 61 d with ^213^Bi to having 8 of 12 rats alive 1 year post-treatment with ^225^Ac [105]. Oliveira-Silva et al. computed cellular S-values for ^211^At, ^225^Ac, ^223^Ra, and their daughters. Unsurprisingly, they found that cumulative S-values for ^225^Ac and ^223^Ra decay chains were 4× greater than the cumulative S-value for ^211^At without including daughter cellular redistribution, explained in that ^211^At emits one α particle per decay compared to the four α particles emitted by ^225^Ac and ^223^Ra decay chains [108].

### 4.3. Auger and Internal Conversion Electron-Specific Radiation Biology Considerations

Auger electrons and some internal conversion electrons have high LET, short path lengths, and high RBE, providing a high radiation dose deposited into small, localized volumes, which shreds DNA with demonstrated therapeutic effect [109,110] and results in apoptotic cell death [111,112,113,114]. Of therapeutic radionuclides, Auger and conversion electron emitters emit the lowest total energy per decay, yet promising Auger emitters release a total energy similar to or slightly less than β^−^-emitters. Specifically, ^193m^Pt (~150 keV/decay), ^195m^Pt (~260 keV/decay), ^237^U (~330 keV/decay), and ^239^Np (~390 keV/decay) release approximately the same energy as ^161^Tb (~250 keV) and ^177^Lu (~180 keV). Though ^76^As emits ~1.5 MeV/decay. Theoretically, Auger electrons allow effective dose delivery to metastatic disease with significant side effect mitigation. In contrast, the ^225^Ac, ^211^At, and ^212^Pb decay chains emit ~34.2 MeV/decay, ~16.1 MeV/decay, and 10.1 MeV/decay, respectively. The production of Auger electron emitters is scalable on low-energy cyclotrons currently distributed across the world [115], and Auger electron emitters primarily decay to stable daughters [16,116]. Compared to α-emitters, both features are advantages of implementing Auger emitters in radiopharmaceutical therapy.

The therapeutic effectiveness of Auger electron radiopharmaceuticals is extremely dependent on subcellular localization and emitted electron spectra [7,14,15,16,17]. Desirable factors include high Auger electron energies, high Auger electron yields, and half-lives (t_1/2_) in the range of hours or days. Additionally, ideal therapeutic radionuclides have low emissions of high-energy photons, as such particles would increase off-target radiation dose and subsequently decrease tumor-to-normal tissue mean absorbed dose rate ratio (TND).

Using a mathematical model to evaluate electron energy and TND, researchers surveyed low-energy electron emitters for small tumor radiopharmaceutical therapy, determining that an ideal radionuclide for small-tumor radiopharmaceutical therapy would have the following likely traits: (1) emits electrons with energy below 40 keV, (2) has a ratio of photon-to-electron energy less than two, (3) decays to a stable daughter nuclide or one with <60 day half-life, (4) has a half-life of 30 min to 10 days, (5) can be produced by proton-, α particle-, deuteron-induced, or neutron capture reactions, and (6) due to difficult labeling chemistries, is not a noble gas. Using this criteria, five radionuclides (^58m^Co, ^119^Sb, ^161^Ho, ^103m^Rh, and ^189m^Os) were recommended, dependent on selective, internalizing targeting vectors [68].

Auger electron emitters have the greatest cytotoxic effect when incorporated into cellular DNA (e.g., via nucleosides like deoxycytidine and deoxyuridine) [15,117,118,119]. Due to Auger electrons’ short pathlengths, increasing the distance between an Auger electron emitter and cellular DNA decreases cytotoxicity [119,120,121,122]. When localized at the cell membrane, ^75^Se-emitted conversion and Auger electrons have RBE equivalent to β^−^-emitters [123]. Conversion electrons have longer path lengths (0.05–13.4 mm) and can yield a therapeutic effect without internalization [112]. For small cell clusters, electrons with energy of 20–30 keV provide an optimal self-absorbed dose within the targeted cell, retaining localized energy deposition [69]. Auger electrons not located in the nucleus can induce damage via indirect mechanisms and non-targeted effects, generating damage beyond the electron range [124]. Using DMSO to inhibit indirect mechanisms, researchers have observed that, depending on the radionuclide, up to 90% of the deposited dose from Auger electron emitters is attributed to indirect mechanisms [125].

In 2008, researchers calculated theoretical dose distributions and MIRD S-values for various Auger-electron-emitting radionuclides and intercellular locations [110]. Resultant dose distributions from ^67^Ga, ^193m^Pt, ^111^In, ^165^Er, ^123^I, ^125^I, ^119^Sb, and ^201^Tl in an 8 mm radius spherical cell with a 6 mm nuclear radius were compared. Of the studied Auger electron emitters, ^119^Sb delivered the highest radiation dose to the cell nucleus when radioactivity was homogenously distributed on the cell surface or within the cytoplasm, and ^193m^Pt delivered highest nuclear dose from nuclear distribution [110]. Other researchers applied Monte Carlo damage simulations and PENELOPE code [126] to calculate biological effectiveness and cellular S-values for the Auger-electron-emitting radionuclides ^125^I, ^119^Sb, ^123^I, ^111^In, and ^99m^Tc. Greater than 75% of DNA with DSBs and SSBs occurred in regions within 2.5 mm of the nucleus center [126].

Living system cell geometry is irregular, motivating exploration of the influence of off-centered nuclei and subcellular localization on subsequently calculated cellular S-values for 12 radionuclides (^123^I, ^125^I, ^119^Sb, ^67^Ga, ^111^In, ^80m^Br, ^89^Zr, ^99m^Tc, ^90^Nb, ^195m^Pt, ^201^Tl, and ^117m^Sn) [109]. As expected, eccentricity greatly impacted S-value calculations for activity distributed on the cell surface, as evidenced by an increase in nucleus-to-cell surface (N←CS) S-values when the nucleus is positioned closer to the cell surface, allowing more low-energy electrons to hit the nucleus and contribute to nuclear dose. Reasonably, these researchers report a decreased cellular nucleus-to-cytoplasm (N←Cy) S-value when the nucleus is off-center, as the displaced cytoplasm pushes radioactivity further away from the nucleus. When distributed on the cell surface, ^67^Ga, ^89^Zr, and ^201^Tl were most impacted by eccentricity, with ^111^In and ^119^Sb least affected [109]. Regarding internal conversion and Auger-electron-emitting radionuclides, it is important to compute dosimetry using Monte Carlo methods and event-by-event simulation to provide enough spatial resolution or else computations underestimate secondary electrons generated by higher-energy conversion electrons, which will greatly underestimate energy deposition [109].

**Figure 6:**
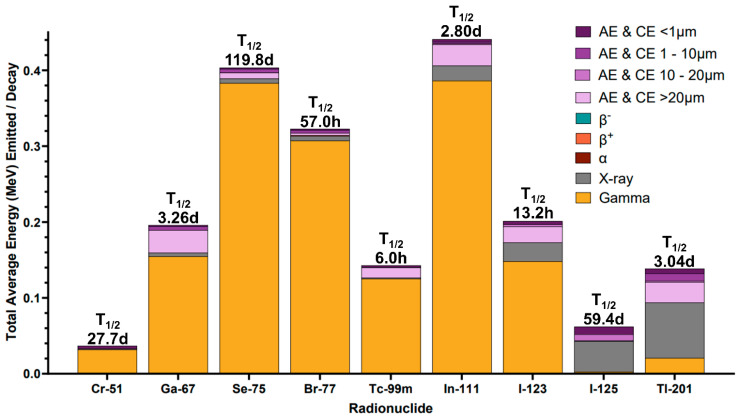
A comparison of total average energy (MeV) emitted by each historically studied Auger-emitting radionuclide per decay, stratified by emission type and Auger and conversion electron range with annotated half-life. Nuclear decay properties are sourced from [76,77].

Of medical radionuclides, almost half are Auger electron emitters. The most common radionuclides historically investigated for Auger electron radiopharmaceutical therapy include ^125^I, ^99m^Tc, ^75^Se, ^77^Br, ^111^In, ^123^I, ^67^Ga, ^201^Tl, and ^51^Cr [11,117,123,127]. Influential studies have used ^125^I, ^99m^Tc, and ^111^In despite them being unideal for Auger electron radiopharmaceutical therapy. Depicted in Figure 6, these radionuclides emit electrons with unideal energies (the greatest proportion electron energy is outside 20–40 keV, ~10–30 μm), produce additional accompanying emissions (e.g., photons for ^99m^Tc and ^111^In), and have half-lives incompatible with radiopharmaceutical production and administration (e.g., ^125^I). Practical issues like access and easy chemical incorporation motivated the study of these unideal Auger electron emitters for therapeutic application. Their lack of induced radiation response when targeting structures besides DNA contributed to a belief that effective Auger electron radiotoxicity is exclusively achieved through nuclear targeting.

**Figure 7:**
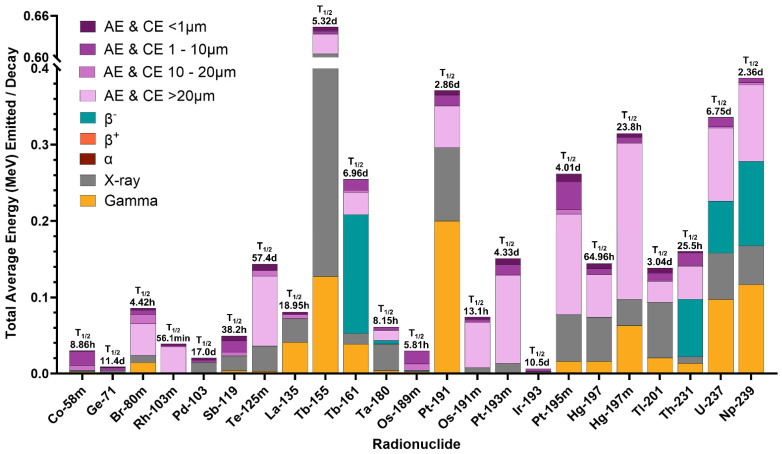
A comparison of total average energy (MeV) emitted by each promising Auger-emitting radionuclide per decay, stratified by emission type and Auger and conversion electron range with annotated half-life. Nuclear decay properties are sourced from [76,77].

Researchers have reliably observed cell toxicity when targeting conversion and Auger-electron-emitting radionuclides to locations besides the cell nucleus, including targeting ^125^I to the cellular membrane [24,128] and ^111^In to the cytoplasm [129]. These documented bystander and non-targeted effects illustrate the complex Auger electron radiobiological response systems, which, though incompletely understood, could be leveraged to promote cell-killing efficacy [16,17,48,125]. Recent efforts by the International Atomic Energy Agency (IAEA) technical meeting—2022 on Auger electron emitters worked to create a scoring system to identify promising Auger-electron-emitting radionuclides. From their dosimetry scoring system, favorable (^71^Ge, ^119^Sb, ^155^Tb, ^180^Ta, ^189m^Os, ^191m^Os, ^191^Pt, and ^193m^Ir) and highly favorable (^125m^Te, ^161^Tb, ^193m^Pt, ^195m^Pt, ^197m^Hg, ^201^Tl, ^231^Th, ^237^U, and ^239^Np) Auger-emitting radionuclides were identified. Relevant to theranostics, their selection system prioritized radionuclides with co-emitted imageable particles for ease of dosimetric evaluation [130]. Figure 7 provides a comparison of emission profiles from various Auger-emitting radionuclides outlined in the literature for promising dosimetry properties.

## 5. Theranostic Considerations

### 5.1. Radiation Dosimetry

Radiopharmaceutical therapy initiates complex radiobiological responses from cells, and a deeper, detailed understanding is required to appropriately harness these effects for the development of greater, more precise cell killing. As outlined in Section 3, researchers have observed differences in cell signaling activation in response to radiation damage to varying cellular structures, as well as a general environment of oxidative stress. Current radiopharmaceutical-specific radiobiology poorly describes both subcellular and macroscopic absorbed dose responses, in part due to a lack of regular application and difficulties in accurately ascertaining in vitro and in vivo dosimetry.

We have shown the wide variation in emissions generated from the same classes of radionuclides, each having implications on the resultant dose distribution profiles. As researchers have shown cell death when targeting radiation dose to locations besides the DNA, subcellular dose distributions have an important impact on cell survival, particularly when considering emissions whose ranges are on the order of a cell diameter (mostly α, Auger, and conversion electrons). Microdosimetry is the study of cellular and subcellular dose distribution and, historically, has relied heavily on computational methods. Many computational studies implement Monte Carlo techniques to predict absorbed dose [109,110,126,131,132]. Microautoradiography [133,134], cell fractionation [135], and microscopy [136,137] measure subcellular radiopharmaceutical distribution, allowing resultant subcellular radiation absorption calculation.

To simplify characterization and calculations, cellular absorbed dose has been commonly modeled as uniformly distributed amongst geometrically simplified spherical cells, representing absorbed dose averages. The uncertainties introduced from these simplifications likely depend on the LET and ranges of emitted particles considered, as well as variability in emissions and decay locations. This is illustrated in an aforementioned study modeling nuclear dose from Auger- and conversion-electron-emitting radionuclides to irregular “off-centered” nuclear geometries [109]. Recent work in experimental microdosimetry utilized immunofluorescent microscopy to track ^212^Pb-labeled antibody kinetics, segment nearby nuclei, and predict the resultant nuclear absorbed dose using GEANT4 Monte Carlo simulations [137]. A comparison of microscopy-based dose calculations against models of uniformly distributed activity in spherical geometries found that the simplified geometry model overestimated nuclear dose contribution from internalized activity and underestimated absorbed dose from the cross-fire of neighboring cells [137]. These two examples illustrate the significant influence of irregular geometry, non-homogeneous activity distributions, and subcellular localization towards absorbed dose from comparatively short-range emissions.

Current clinical practices apply a fixed administered radioactivity in radiopharmaceutical dosing, similarly to chemotherapy agents, in which standard doses are determined from toxicity profiles from early phase clinical trials [138,139], with some retrospective analysis. A patient with low receptor expression would resultantly be underdosed. In clinical applications, routine application of theranostics and dosimetry can aid patient selection (by molecular imaging phenotyping and target expression determination), dose optimization, post-treatment dose mapping, treatment monitoring and adjustment [140]. On the cellular level, software tools including MIRDcell [141] or modifications to traditional EBRT biological response modeling [142] can help, but great care needs to be taken to minimize uncertainties by accurately determining administered and time-integrated activities [142], which can be a technical challenge with low energy emissions and low activity levels. Elementally matched, theranostic pairs provide a great advantage in overcoming low energy emission detection and in vivo patient dosimetry. Greater dosimetry characterization is necessary and will be heavily dependent on the development of accurate theranostic pairs. With the development and expansion of radiometals in radiopharmaceutical application [143], greater options exist for selecting appropriate imaging and therapeutic radionuclidic pairs.

Historically, many computational dosimetry studies have emphasized the dosimetric advantage of implementing therapeutic radionuclides, which have minimal co-emitted photon contaminants, commonly discussed in terms of an emitted photon-to-electron ratio [68]. High-energy photon emissions provide an additional off-target radiation dose, increase the dose to healthy tissue, and potentially decrease the therapeutic window between tumor control probability (TCP) and normal tissue complication probability (NTCP). However, with increased concern for the impact of differences in biological distributions between therapeutic radionuclides and non-isotopic theranostic imaging pairs, as well as the growing need for personalized dosimetry, researchers have re-visited the advantage of applying therapeutic radionuclides which have imageable co-emissions [130]. Post-^177^Lu radiopharmaceutical therapy, SPECT imaging records the exact biological distribution of the therapeutic radionuclide for dosimetry. If implemented as a large-scale routine clinical practice, these studies might explain variations in absorbed dose responses reported in various clinical trials.

EBRT implements a prescribed tumor-absorbed dose strategy, which, if applied to radiopharmaceutical therapy, would require pre-treatment administration of a PET/SPECT imaging surrogate to measure patient-specific tumor receptor expression and trace biological clearance. A proposed workflow includes using serial, quantitative imaging to measure the tumor and organ time–activity distributions of an imaging surrogate. After extrapolating characterized differences in radiological and biological half-lives to the therapeutic analog, this data allows the prediction of absorbed dose, modeling of biologically effective dose, and the optimization of administered therapeutic activity [139]. PET and SPECT imaging each have advantages and disadvantages, and the choice of modality will likely depend on the decay characteristics of the chemically matched theranostic pair.

### 5.2. PET Imaging

Proton-rich nuclei decay by either internal conversion, electron capture, or positron (β^+^) emission. Following decay and subsequent emission, the β^+^ will travel through matter, losing energy via collisions until annihilating with an electron, its antimatter counterpart. Annihilation converts particle rest masses into two antiparallel photons with energy of 511 keV [144,145]. For PET imaging, rings of detectors (windowed for energies between 350 and 650 keV and time delays of 3–6 ns) identify annihilation coincidence photons [144], indicating that (β^+^) annihilation events occur along the line of response (LOR) drawn between two detectors. Time-of-flight (TOF) PET measures delay between the annihilation photon’s detection, more precisely determining the annihilation event’s location along the LOR [144]. Statistical reconstruction or filtered back projection of the coincidence data recovers the PET image’s quantitative volumetric activity distribution [144]. Importantly, PET images comprise the distribution of annihilation events (not the β^+^ decay sites) recorded by the scanner, considering and filtering noise from scatter (the detection of coincidences post-Compton scattering of one or more annihilation photons) and random events (the simultaneous detection of two photons at 350–650 keV not stemming from the same annihilation) [144]. The β^+^-emitting radionuclide decays at the radiopharmaceutical site, and the fundamental limit of spatial resolution, which can be achieved through PET imaging, is primarily calculated from the distance the β^+^ travels before annihilation. Emission of a higher-energy β^+^ results in poorer spatial resolution, which minimally is on the order of millimeters. Because of conservation of momentum, annihilation photons are not emitted from the decaying radionuclide perfectly antiparallel, which results in angles slightly smaller than 180°. Scanners assume a perfectly antiparallel LOR, which results in additional uncertainty in the placement of annihilation events and greater non-collinearity impacts for larger-diameter detector ring sizes. Innate detector spatial resolution and non-collinearity also contribute to PET image resolution limits [145]. With proper calibration and characterization, PET imaging sensitivities range between 2 and 10% (0.02–0.1 CPS/Bq) [9] and provide invaluable physiological information [144].

Traditional PET imaging implements a ~20 cm field of view (FOV). Improvements in electronic signal handling and detector fabrication have allowed the production of total-body PET scanners with a 200 cm FOV. The increased FOV not only allows simultaneous collection of radiopharmaceutical distribution throughout the entire body but also dramatically increases detector sensitivity (~40×) and signal-to-noise ratio, providing higher-quality images at lower β^+^ emission levels [146,147]. One implication of higher-sensitivity PET imaging is the ability to image radiopharmaceuticals at longer time points. Therapeutic radionuclides commonly have half-lives on the order of a week, and extended time-point imaging of corresponding imaging radionuclides with shorter half-lives is advantageous. More interestingly, higher-sensitivity PET will allow clinically relevant imaging with a broader range of radionuclides that have smaller β^+^ branching ratios. This opens opportunities within theranostics for imaging previously un-imageable therapeutic radionuclides and provides a wider pool of potential diagnostic imaging pairs.

### 5.3. SPECT Imaging

In SPECT imaging, a gamma detector with a multihole collimator provides 2D spatial correlation of detected photons, allowing emission location. Collecting projections of these superimposed datasets allows 3D differentiation [148]. Preclinical SPECT scanners provide sub-millimeter spatial resolution [149], and the choice of SPECT collimator significantly impacts scanner capabilities. Images produced using pinhole collimators achieve higher resolution while sacrificing detector efficiency [149]. The problems with pinhole collimation have mostly been solved by using iterative reconstruction methods, correcting for attenuation, scatter, and response function [148,149]. Most SPECT scanner detectors and components are tuned to maximize the accurate detection of ^99m^Tc’s 142 keV emission [148], with sensitivity 1/200th of that of PET systems [9]. By reconstructing a SPECT image with different energy windows corresponding to emissions from different radionuclides, multi-radionuclide SPECT imaging can be accomplished. Researchers simultaneously measured similar biological distributions from co-injected [^161^Tb]Tb-DOTA-LM3 and [^177^Lu]Lu-DOTA-LM3, supporting previous assumptions on the chemical similarities of ^177^Lu and ^161^Tb [150]. In preclinical imaging, ^111^In and ^67^Ga have also been simultaneously distinguished [151].

## 6. Methods and Techniques Employed to Understand Radiation Mechanisms

### 6.1. Clonogenic Assays

First reported in 1956, clonogenic assays determine the reproductive potential of cells [152] and are particularly useful in measuring toxicity responses to agents, treatments, and common radiation. In general, a known number of cells are exposed to a test agent and plated at low concentrations such that, after a length of time allowing surviving cells to replicate (e.g., 2 weeks), cells are fixed, stained, and colonies of greater than 50 cells are counted. Normalization of counted test colonies to untreated control values allows the calculation of survival fractions and subsequent generation of dose–response survival curves [153]. These linear quadratic survival curves primarily characterize targeted cell response and change in shape based on cell-killing efficacy [17]. Higher LET radiation results in a straighter, steeper curve with less shoulder, indicating a higher ratio of lethal lesions [4]. Because clonogenic assays are time intensive and low throughput, researchers have explored the ability of MTT and luminescence viability assays to replace or compliment characterization by clonogenic assay. It is important to note that clonogenicity and viability are not the same metric, so viability assays can overestimate survival fractions [154,155,156].

### 6.2. Comet Assays

First reported by Ostling and Johanson in 1984, the highly sensitive comet assay uses electrophoresis to separate and characterize DNA fragmentation [157] from DNA with SSBs and DSBs. Generally, irradiated or agent-exposed cells are suspended as single cells in low-melting-point agarose (0.5–1%) and cast onto fully frosted microscope slides. Embedded cells are then lysed through slide submersion in neutral or alkaline lyse buffers and washed before electrophoresis processing. Because alkaline conditions separate and unwind DNA strands, alkaline comet assays are required for SSB measurement in addition to DSBs [158,159]. Electrophoresis voltage and time are dependent on electrophoresis buffer concentration and the extent of DNA damage. During electrophoresis, DNA fragments will travel away from the cell nucleus towards the anode as a function of fragment size and charge, and fluorescent stains are used to visualize DNA fragment migration. The distance that DNA fragments travel from the core of the nucleus (the length of the comet tail) is related to the extent of DNA damage [159]. Comet tails can be scored visually or through automated image analysis with comparable results, but conversion of the score to a unit (e.g., breaks per cell, breaks per Da9, or dose in Gy) through calibration curves is critical [160]. One limitation of the comet assay is the difficulty of comparing results between studies and institutions due to assay variability, which persists with calibration [160]. For example, the assay’s sensitivity to salt concentration can cause heterogenous tail lengths across the same slide if salt gradients exist [159]. The development of international comet assay workshops and a standardized protocol for ex vivo adaptation [158] has increased consistency [161].

### 6.3. Immunofluorescent Microscopy

In early cell response to DSB DNA damage, serine-139 on H2AX histones in regions surrounding the break are phosphorylated, markers referred to as γH2AX foci [162]. This chromatin modification is used to signal DSBs, stabilize broken DNA ends, increase DNA accessibility by decreasing chromatin density, and recruit DNA repair proteins [163]. γH2AX foci formation begins minutes after exposure to ionizing radiation and peaks in quantity at 30 min [164]. Researchers consider each foci to represent a DSB, and quantification of γH2AX foci using immunofluorescent microscopy provides a reliable method for spatial and temporal quantification of DSB formation and repair [163]. Cells within the S-phase, which are actively synthesizing new DNA, produce a more dispersed, less concentrated pattern of γH2AX foci formation in response to DSBs [165]. The DNA damage response regulator protein ATR can produce phosphorylated γH2AX during DNA replication, providing another issue when quantifying DSBs in S-phase cells [164].

After exposing cells to ionizing radiation or a test agent for DSB formation, cells are fixed, permeabilized to allow entrance of immunofluorescent agents into the cell, and blocked for non-specific antibody uptake. Next, cells are incubated with a primary antibody for γH2AX (ex: mouse anti-γH2AX antibody) at 4 °C. The next day, the primary antibody is removed, cells are washed with a low percentage blocking solution, and cells are incubated with an appropriate secondary fluorescent antibody (ex: goat anti-mouse). After a final washing, cells are mounted with mounting medium and a coverslip to prevent an air bubble embedding during hardening before sample analysis using fluorescent microscopy [165]. By incorporating appropriate combinations of primary and secondary antibodies, markers of DNA repair beyond γH2AX foci can be simultaneously monitored [165,166]. Examples include 53BP1, which triggers DSB repair via non-homologous end joining, BRCA1, which stimulates end resection and homologous recombination [167], RAD51, another marker of homologous recombination [168], and PARP1, a first responder to initiate DNA repair via homologous recombination [166].

### 6.4. Computational Monte Carlo Dosimetry

The absorbed radiation dose is the energy absorbed from radiation by matter (defined for a given volume and mass). As described earlier, interactions of charged and uncharged particles with matter represent a complex system dependent on multiple stochastic processes. Exact mathematical modeling of particle transport through matter is difficult to solve analytically, and numerical solutions quickly become computationally heavy with increased geometry and system complexity. Because many features of radiation and radiation interactions with matter are probabilistic, modeling particle absorption and transport through matter is an ideal application for Monte Carlo simulation. The Monte Carlo method uses many iterations of randomly generated numbers to numerically sample probability distributions and predict large-scale interactions and outcomes. Common code systems and toolkits for Monte Carlo simulation of radiation transport include MCNP [169], EGS [170], Geant4 [75], and PENELOPE [171], with many options for adaptations to internal dosimetry [172,173,174,175,176,177,178,179,180] and FDA-cleared software packages [181]. More thorough reviews discussing advantages and limitations are available [181,182,183].

### 6.5. Medical Internal Radiation Dose (MIRD) Schema

The MIRD schema for determining internal dosimetry uses time-integrated activity of internally distributed radioactive sources and a source–target region approach, providing mathematical methods, anatomical and biological models, and nuclear decay data. The MIRD strategy can be adapted to multiple levels of biological organization, including the whole body, organs, organ and tissue subregions, cells, and subcellular compartments. For the chosen biological model, source regions (areas containing the radionuclide) and target regions (where the absorbed dose is being calculated) are determined, and S-values are calculated dependent on radionuclidic decay information, the biological model, and the source/target pair. For a particular target region, the absorbed dose rate delivered to the region is computed by summing the product of all activities within source regions and corresponding S-values. The society of nuclear medicine and molecular imaging (SNMMI) MIRD committee continues updating methods, models, mathematical formulas, assumptions, and nuclear data [77,184,185], including the community platform MIRDSoft, which supports and distributes a variety of MIRD-based software packages [186]. Commonly used for human internal dosimetry calculation, the OLINDA/EXM software implements the MIRD technique [187,188]. More thorough reviews discussing advantages and limitations are available [181,182,189].

## 7. Summary and Perspective

Therapeutic radiopharmaceuticals elicit complex radiobiological responses similar to yet distinctly different from the more widely studied EBRT responses. In clinical applications, EBRT is well studied and precisely executed, with features extremely different from radiopharmaceutical therapy, in which dose deposition is driven by radionuclide decay properties and radiopharmaceutical distribution. It is necessary to fill the gap in understanding radiopharmaceutical-specific radiation biology to continue driving the development of this promising technology. Theranostic radiopharmaceuticals and their application in dosimetry can help fill this gap from the clinical level, and thoughtful microdosimetry-driven study into the therapeutic potential of characteristic emissions from a broad, diverse range of radionuclides will help fill the gap on the cellular response level. Challenges exist in broad clinical and preclinical implementation of standardized techniques for dosimetry determination and response characterization. Additionally, limited production, availability, and chemical incorporation strategies for novel radionuclides exist, with the need for ever-broadening development of new disease-targeting molecules.

Low-LET, β^−^-emitting radionuclides control tumor growth in advanced disease and currently lead in clinical application. As with Auger and conversion electron emitters, special consideration of the unique emitted electron spectra of each radionuclide is essential to a comprehensive understanding of their radiobiology. Of all radioactive decay modes, α-emitters produce the highest LET, highest RBE particles and can overcome resistance to other radiopharmaceutical therapies. However, α-emitters are frequently associated with problematic toxicity in clinical trials and are harder to produce at scale. Finally, Auger and conversion electron emitters decay to stable daughters and have scalable production via low-energy proton cyclotrons, but their absorbed doses are much more sensitive to subcellular location. High-LET radiation from α-emitters and Auger and conversion electron emitters is absorbed within a short range of the decay site and, compared to low-LET radiation, induces more complex DNA damage, higher off-target effects, greater cellular toxicity, and is less impacted by hypoxia. These targeted and non-targeted bystanders, and abscopal effects are dependent on LET, dose, dose rate, dose fractionation, and additionally overlooked and poorly understood viables in the tumor microenvironment. Careful selection of radionuclides for specific applications will be required.

A wide variety of assays and technologies have historically been implemented for radiation biology research, with notable innovations. Gold-standard assays, including the clonogenic and comet assays, can be time consuming, low throughput, and vary between institutions, and significant efforts in standardization to decrease variability and exploration of additional, complimentary cell proliferation and viability assays are ongoing. Innovation into whole-body PET imaging allows capturing longer time-points, as well as imaging a broader range of β^+^-emitters with low branching ratios, and more routine application of SPECT imaging will help increase understanding of absorbed dose response in ^177^Lu clinical treatments. Additional development of clinical standards to quantify and measure side effects will also further radiopharmaceutical development.

Theranostic radiopharmaceuticals have dramatic potential to improve clinical treatments for a variety of diseases. Accurate detection and elimination of diseased cells will require careful design and tailoring of radiopharmaceuticals including the biological targeting vector, radionuclide, linkers, chelators and radionuclide incorporation strategy—an ever-broadening toolkit of components, which could allow drug tuning for precision medicine. Careful study of radiopharmaceutical-specific radiobiological responses, as well as an increased use of theranostics to accurately characterize dose-dependent responses in disease systems, is necessary to help realize the full potential of radiopharmaceutical therapy and ensure that theranostics become standard of care treatments for many diseases in the 21st century.

## Figures and Tables

**Table 1:** A summary of differences between external beam radiation therapy (EBRT) and radiopharmaceutical therapy (RPT) with potential implications. Inspiration from [17].

	EBRT	RPT	Potential Implications for RPT
Range	Long (~3% of 6 MV photons attenuated per cm traveled)	Short (<µm Auger, 10’s of µm α) to medium (mm β^−^)	When delivered to diseased cells, shorter-range radiation would result in less off-target radiation dose with minimized side effects. This would increase the therapeutic window, allowing administration of a higher, more cytotoxic radiation dose and potentially better treatment outcomes.
LET	Extremely low	High (Auger, α) and low (β^−^)	Delivered to the right location, high-LET radiation increases cytotoxicity and provides more lethal cell killing.
Particle Type and Energy	Uniform	Varied in type and energy according to radionuclide	Varied particle types and energies can elicit a combination of differing responses, which increases uncertainty in understanding induced effects. Thoughtful, careful study of these differences is needed to effectively tailor combinations of administered radiation towards desired outcomes.
Radiation Field	High, unidirectional fluence	Sparce, omnidirectional	The sparce, omnidirectional radiation field generated from radiopharmaceutical emissions provide more challenging dosimetry calculations, particularly when considering the cellular and subcellular scales.
Dose Rate	High (>1 Gy/min)	Low (0.1–1 Gy/h) and continuous according to radiological and biological half-life	A low dose rate could allow repair of sublethal damage, yet a low dose rate could induce a chronic environment of oxidative stress.
Absorbed Dose	Homogenous across tumor	Heterogenous due to varied tissue perfusion and receptor expression	Heterogenous dose distributions can account for increased uncertainty in observed absorbed dose responses, a poorly understood metric for RPT.
Relative Biological Effectiveness	Low	High (Auger, α) and low (β^−^)	High RBE from high-LET RPT results in more effective cell killing.
DNA Damage	Low complexity	High complexity (Auger, α) and low complexity (β^−^)	High-LET radiation induces more complex DNA damage, which is more difficult to repair and causes greater cell killing.
Tumor Cell Cycle Sensitivity	High, mitigated with fractionation	Potential synchronous effects	Potential G2/M cell cycle arrest due to significant complex DNA damage.
Targeted Effects	Primarily indirect mechanism	Option for primarily direct (Auger, α) or indirect mechanisms (β^−^)	Direct mechanisms of targeted damage are less susceptible to ion recombination or antioxidant neutralization, making direct mechanisms more lethal.
Impact of Hypoxia	Significant	Low for high-LET	Hypoxia increases radiation resistance, so high-LET radiation advantageously experiences less hypoxia-induced resistance.
Common Fractionation Schedules	Daily	Multiweek	Unoptimized, multiweek treatment schedules could be more or less convenient for patients.
Dosimetry Characterization	Personalized, prospective, and well defined	Standard dosing, little retrospective and poorly understood analysis. Many agents require a theranostic imaging pair.	Poorly understood dosimetry and non-clinically standardized, irregular dosimetry characterization likely contribute to uncertainties in measuring and understanding RPT absorbed dose response.

**Table 2:** Energy deposition in tissue for therapeutic decay properties. Adapted from [35] with RBE values from [15,70,71,72].

Emission	Particles	Multiplicity	*E*_(__min)_–*E*_(__max)_	Range	LET	RBE
α particle	Helium nuclei	1	5–9 MeV	40–100 µm	~80 keV/μm	~20
β^−^ particle	Energetic electron	1	0.050–1.3 MeV	0.05–6.2 mm	~0.2 keV/μm	~1
Conversion & Auger electrons	Low energy electrons	5–30	eV–2.7 MeV	2 nm–13.4 mm	~2–26 keV/µm	~5–20

**Table 3:** Comparison of average β^−^ emissions from prominent preclinically and clinically applied therapeutic radionuclides. Nuclear decay properties are sourced from [76,77], and electron ranges approximated by fitting R_CSDA_ from NIST [73] and GEANT4 Monte Carlo simulation [75].

Radionuclide	Half-Life	Prominent Electron Emissions (*I*_B_)	Approx. CSDA Range in H_2_O (µm)
^90^Y	64.05 h	β^−^ = 932.4 keV (0.99985)	4300
^177^Lu	6.64 d	β^−^ = 148.8 keV (0.7944)	270
^161^Tb	6.89 d	β^−^ = 137.7 keV (0.257)	240
		157.4 keV (0.65)	300
		AE = 5.16 keV (0.879)	1.3
		CE = 16.6 keV (0.41)	6.3
		39.8 keV (0.424)	30

## Data Availability

No new data were created or analyzed in this study. Data sharing is not applicable to this article.
